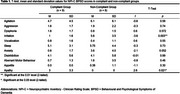# Exploring the Impact of Bright Light Therapy on Sleep and Behavioural Symptoms in Dementia: An Analysis of Patient Compliance

**DOI:** 10.1002/alz70861_108434

**Published:** 2025-12-23

**Authors:** Shivani Patel, Marianne T Elegores, Sarah Elmi

**Affiliations:** ^1^ Ontario Tech University, Oshawa, ON Canada; ^2^ Ontario Shores Centre for Mental Health Sciences, Whitby, ON Canada; ^3^ University of Toronto, Toronto, ON Canada

## Abstract

**Background:**

Bright light therapy (BLT) is an established intervention for mood and sleep disorders through its regulation of circadian rhythms. However, its effects on the behavioural and psychological symptoms of dementia (BPSD) are less well understood and remain an area of ongoing investigation.

**Method:**

Participants diagnosed with dementia were recruited from the Geriatric Transitional Unit at Ontario Shores Mental Health Sciences. Each participant received BLT at an intensity of 10,000 lux for 30 minutes each weekday over a four‐week period. Physiological parameters such as heart rate and electrodermal activity were collected using Empatica E4 wristbands, while BPSD assessments, using the Neuropsychiatric Inventory‐Clinician rating scale, were conducted at three time points: prior to the intervention, at the end of the four‐week intervention, and four weeks following the completion of therapy. Participants were categorized into compliant and non‐compliant groups based on their participation in the therapy sessions. Between‐group analyses were conducted using two‐tailed t‐tests.

**Result:**

Participants in the compliant group (n = 9) demonstrated longer sleep durations and greater overall improvements in sleep measures compared to those in the non‐compliant group (n = 8). Significant differences were observed between groups in irritability (p = 0.003) and apathy (p = 0.021) scores. Specifically, the compliant group exhibited lower levels of irritability (M = [1.0], SD = [1.6]) compared to the non‐compliant group (M = [5.6], SD = [3.5]), but higher levels of apathy (M = [3.0], SD = [3.3] versus M = [0.0], SD = [0.0]). Notably, heart rate variability (HRV), measured as the root mean square of successive differences, was found to be lower in the compliant group, but the difference was not statistically significant.

**Conclusion:**

Compliance with BLT was associated with improved sleep outcomes in patients with dementia. The findings suggest that lower levels of irritability and higher apathy may serve as potential predictors of adherence to BLT. These factors should be considered when identifying appropriate candidates for light‐based interventions in the context of dementia care and improving sleep.